# Monitoring peripheral neutrophil and T-lymphocyte subsets could assist in differentiating the severity and disease progression of coronavirus disease 2019

**DOI:** 10.18632/aging.202701

**Published:** 2021-03-19

**Authors:** Liye Meng, Jiao Xu, Junlong Zhang, Keyi Zhang, Pengcheng Shang, Qingfeng Li, Yi Li, Lanlan Wang, Bei Cai

**Affiliations:** 1 Department of Laboratory Medicine/Research Center of Clinical Laboratory Medicine, West China Hospital, Sichuan University, Chengdu, China; 2 Department of Laboratory Medicine, Public Health Clinical Center of Chengdu, Chengdu, China

**Keywords:** coronavirus disease 2019, COVID-19, lymphocytes, CD3+CD4+ T cells, neutrophil to CD4+ T lymphocyte ratio/N4R

## Abstract

Helper T cells (CD3+CD4+ T cells) and cytotoxic T cells (CD3+CD8+ T cells) play direct and indirect antiviral roles. This study retrospectively explored the clinical significance of peripheral lymphocytes, especially the dynamic analysis of T-cell subsets, in determining coronavirus disease 2019 (COVID-19) severity and progression. Seventy-nine patients with COVID-19 in the Public Health Clinical Center of Chengdu from January to February 2020 were included, 59 of which were analyzed for dynamic peripheral T-cell subsets expression. The neutrophil to CD4+ T lymphocyte ratio (N4R) and neutrophil to CD3+ T lymphocyte ratio (N3R) showed clinical significance in differentiating severe or critically-severe COVID-19, with area under receiver operating characteristic curves (AUCs) of 0.933 and 0.900, respectively (P < 0.05). COVID-19 patients with more baseline peripheral lymphocytes or NK cells were prone to test negative to severe acute respiratory syndrome coronavirus 2 (SARS-CoV-2) after therapy (P < 0.05), and the AUC of NK cells for predicting negative results of SARS-CoV-2 RNA detection after therapy was 0.800. When the number of peripheral CD3+CD4+ and CD3+CD8+ T cells in COVID-19 patients continuously increased 6-9 days after baseline, the period of disease exacerbation could be delayed for more than 2 weeks after admission. Baseline N4R and N3R could be potential biomarkers for assisting in differentiating COVID-19 severity, and dynamically monitoring peripheral CD3+CD4+ and CD3+CD8+ T cells 6-9 days after baseline could help clinicians to evaluate disease progression in COVID-19 patients.

## INTRODUCTION

Coronavirus disease 2019 (COVID-19) is an infectious disease caused by severe acute respiratory syndrome coronavirus 2 (SARS-CoV-2). COVID-19 has rapidly spread globally and become a serious public health problem since its outbreak in December 2019. To date, 223 countries/regions have been affected by this epidemic, and over 102 million people have been confirmed to have COVID-19, while more than 2 million have died. COVID-19 patients may have symptoms similar to the common cold or influenza, such as fever, cough, and fatigue, but their condition progresses quickly. Severe patients can appear short of breath and develop acute respiratory distress syndrome or organ failure, and some even die [1–3]. Therefore, monitoring COVID-19 severity is helpful in clinical decision making.

Laboratory medicine plays a critical role in COVID-19 diagnosis. Detecting nucleic acid and serum specific antibodies of SARS-CoV-2 is equally important in the diagnosis of COVID-19, according to the Diagnosis and Treatment Protocol for Novel Coronavirus Pneumonia (Trial Version 7) [4]. Because of the critical roles of neutrophils and lymphocytes in viral immunity, more attention has been paid to the clinical significance of peripheral neutrophils and lymphocytes, especially T-lymphocyte subsets, in this study. T lymphocytes, the key lymphocytes in adaptive immunity, include helper T cells (CD3+CD4+ T cells) and cytotoxic T cells (CD3+CD8+ T cells). CD3+CD8+ T cells play a direct cytotoxic role in the antiviral process, while CD3+CD4+ T cells assist other immune cells in activation, such as cytotoxic T cells and B cells. Wölfel et al. [5] demonstrated that SARS-CoV-2 replication was more active in the early stage of COVID-19, and a rapid decline in the SARS-CoV-2 load could not be observed after seroconversion. Therefore, early protection and antiviral immune function may be dependent on humoral and cellular immunity. Focusing on peripheral lymphocyte subsets can be helpful to understand the disease state and progression of COVID-19. Lymphopenia has been recognized as a characteristic of patients with COVID-19 [1, 4, 6], especially in severe cases [7, 8]. However, studies on the application and significance of lymphocyte subsets in COVID-19 are rare. This study retrospectively analyzed and explored the clinical application of peripheral neutrophils and lymphocyte subsets, especially neutrophil to lymphocyte subsets ratio and the dynamic analysis of T-cell subsets, in auxilarly determining COVID-19 severity and progression.

## RESULTS

### Basic information and laboratory characteristics of patients with COVID-19

A total of 79 patients were involved in this study, including 41 males and 38 females, and the male patients were significantly younger than the female patients (P = 0.041). The average age had an upward trend with the worsening of COVID-19 (P = 0.004). In the mild and moderate group, condition of four patients (4/56, 7.14%) deteriorated, while in the severe and critically-severe group, that of three patients worsened, and one died (4/23, 17.39%). Additionally, the proportions of exacerbated or fatal cases in mild, moderate, severe, and critically-severe patients were 0%, 9.3%, 16.67%, and 18.18%, respectively. The percentage of improvement within 1 week of admission in mild or moderate COVID-19 patients (22.22%) was twice that in severe or critically-severe patients (11.76%) (Table 1). Table 2 shows that 54.43% (43/79) of COVID-19 patients had a history of going to or living in Wuhan or coming into contact with people from Wuhan. Fever and cough were the most common symptoms in all COVID-19 patients. Over 90% of the severe or critically-severe COVID-19 patients had bilateral pneumonia.

**Table 1:** Basic information and baseline laboratory indicator characteristics of patients with COVID-19.

		**Mild and moderate group** **(n = 56)**		**Severe and critically-severe group(n = 23)**		**P value**
		**Mild** **(n = 14)**	**Moderate** **(n = 42)**	**Severe** **(n = 12)**	**Critically-severe** **(n = 11)**	
Gender						
Male		8	19	7	7	0.635
Female		6	23	5	4	
Average age (y)		40.14±15.94	51.00±16.26#	57.25±18.26#	64.09±15.04#∆	0.004
Outcome						
Improved (n, %)		7 (50%)	20(46.51%)	9 (75%)	8 (72.73%)	0.042
Stable (n, %)		7(50%)	18 (44.19%)	1 (8.33%)	1(9.09%)	
Exacerbated (n, %)		0	4(9.30%)	2 (16.67%)	1 (9.09%)	
Dead (n, %)		0	0	0	1 (9.09%)	
Follow-up period						
Improved	Within 7 days	6 (22.22%)		2 (11.76%)		0.770
	8-14 days	14 (51.85%)		10 (58.82%)		
	>14 days	7 (25.93%)		5 (29.41%)		
	
Exacerbated or dead	Within 7 days	4		0		0.029
	8-14 days	0		4	
Leukocytes (×10^9^/L, normal range: 3.5-9.5)^a^		6.00±2.10		7.11±2.78		0.122
Increased, n/N (%)		5/46(10.87%)		2/13(15.38%)		0.643
Decreased, n/N (%)		5/46(10.87%)		0/13(0.00%)		0.576
Neutrophils (×10^9^/L, normal range: 1.8-6.3)^a^		4.18±1.92*		5.74±2.52		0.019
Increased, n/N (%)		5/46(10.87%)		4/13(30.77%)		0.097
Decreased, n/N (%)		2/46(4.35%)		0/13(0.00%)		1.000
Lymphocytes (×10^9^/L, normal range: 1.1-3.2)^b^		1.33±0.58*		0.88±0.48		0.013
Increased, n/N (%)		0/46(0.00%)		0/13(0.00%)		/
Decreased, n/N (%)		18/46(39.13%)		9/13(69.23%)		0.054
NLR^b^		2.90 (2.11~4.84)*		5.44(4.20~14.99)		0.001
Monocytes (×10^9^/L)^a^		0.42±0.20		0.41±0.21		0.904
Increased, n/N (%)		7/46(15.22%)		3/13(23.08%)		0.676
Decreased, n/N (%)		0/46(0.00%)		0/13(0.00%)		/
neutrophils/monocytes^b^		10.35(8.10~13.66)*		14.65(11.42~18.64)		0.005
Lymphocytes/monocytes^a^		3.58±1.69*		2.36±1.06		0.017
Eosinophil (×10^9^/L)^b^		0.05(0.01~0.07)		0.01(0.01~0.10)		0.507
Basophil(×10^9^/L)^b^		0.02(0.01~0.02)		0.02(0.01~0.02)		0.800
Erythrocytes(×10^12^/L)^a^		4.42±0.54*		3.97±0.86		0.023
Hemoglobin (g/L)^a^		131.43±17.55*		116.31±27.66		0.020
Hematocrit (L/L)^a^		40.57±4.92*		36.24±7.28		0.015
Coefficient of variation of RBC distribution width (RDW-CV) (%)^a^		12.58±0.58		13.12±1.17		0.125
Platelet (×10^9^/L)^a^		197.87±65.91		185.85±75.12		0.575
CD3+ T lymphocytes (cells/μL, normal range: 941-2226)^b^		713.50 (506.00~1204.25)*		455.50(279.50~644.75)		0.005
Decreased, n/N (%)		25/40(62.50%)		14/16(87.50%)		0.107
CD3+CD4+ T lymphocytes (cells/μL, normal range: 471-1220)^b^		397.00 (271.25~698.25)*		234.00(100.00~411.00)		0.004
Decreased, n/N (%)		23/40(57.50%)		13/16(81.25%)		0.094
CD3+CD8+ T lymphocytes (cells/μL, normal range: 303-1003)^b^		266.00 (180.50~411.00)*		197.00(108.75~248.00)		0.032
Decreased, n/N (%)		24/40(60.00%)*		14/16(87.50%)		0.047
CD4/CD8 ratio^a^		1.78±0.88		1.33±0.63		0.068
B lymphocytes (cells/μL, normal range: 175-332)^b^		90.50 (59.75~135.25)		64.00(33.50~128.75)		0.215
Decreased, n/N (%)		27/32(84.38%)		9/10(90.00%)		1.000
NK lymphocytes (cells/μL, normal range: 154-768)^b^		112.50(84.50~163.50)		115.00(50.75~189.25)		0.658
Decreased, n/N (%)		24/32(75.00%)		7/10(70.00%)		1.000
N3R^b^		4.08 (3.19~6.70)*		13.21(8.90~31.62)		0.002
N4R^b^		6.66 (5.26~10.58)*		41.55(16.58~87.20)		0.001
N8R^b^		12.20 (8.83~24.32)*		26.74(18.52~52.64)		0.022

Note: ^a^described as mean ± s; ^b^described as median and interquartile; # compared with mild patients, P < 0.05; ∆ compared with moderate patients, P < 0.05; *compared with severe and critically-severe group, P < 0.05; NLR: neutrophil to lymphocyte ratio; N3R: neutrophil to CD3+ T lymphocyte ratio; N4R: neutrophil to CD3+CD4+ T lymphocyte ratio; N8R: neutrophil to CD3+CD8+ T lymphocyte ratio.

**Table 2:** Clinical information and characteristics of patients with COVID-19.

	**Mild and moderate group (n = 56)**		**Severe and critically-severe group (n = 23)**		**P value**
	**Mild** **(n = 14)**	**Moderate** **(n = 42)**	**Severe** **(n = 12)**	**Critically-severe** **(n = 11)**	
Exposure history within past 14 days*, No. (%)	28 (50.00%)		15 (65.22%)		0.217
Symptoms, No. (%)					
Fever	39 (69.64%)		21 (91.30%)		0.041
Cough	34 (60.71%)		15 (65.22%)		0.708
Short of breath	8 (14.29%)		7 (30.43%)		0.119
Myalgia or fatigue	8 (14.29%)		4 (17.39%)		0.738
Chest x-ray and CT findings					0.010
Unilateral pneumonia	21 (37.50%)		2 (8.70%)		
Bilateral pneumonia	35 (62.50%)		21 (91.30%)		
Treatment					
Oxygen inhalation	35 (62.50%)		20 (86.96%)		0.032
Glucocorticoid	0 (0.00%)		2 (8.70%)		0.082
Antiviral treatment	54 (96.43%)		13 (56.52%)		<0.001
Interferon atomization	50 (89.29%)		21 (91.30%)		1.000
Thymalfasin	15 (26.79%)		13 (56.52%)		0.012
Coexisting disorders, No. (%)					
Hypertension	4 (7.14%)		6 (26.09%)		0.031
Diabetes	4 (7.14%)		4 (17.39%)		0.221
Coronary heart diseases	2 (3.57%)		3 (13.04%)		0.145

Note: *Exposure history indicated the patients had gone to or lived in Wuhan or had come into contact with people from Wuhan.

Table 1 demonstrates that 45.76% of COVID-19 patients were characterized by lymphopenia, and among them, the proportion of patients with strikingly decreased T, B, and NK cells was 69.64%, 85.71%, and 73.81%, respectively. About 15-16% of cases also manifested an increase in baseline peripheral neutrophils or monocytes. A marked increase in neutrophils, the neutrophil to lymphocyte ratio (NLR), the neutrophil to monocyte ratio (NMR), the neutrophil to CD3+ T lymphocyte ratio (N3R), the neutrophil to CD3+CD4+ T lymphocyte ratio (N4R), and the neutrophil to CD3+CD8+ T lymphocyte ratio (N8R) and a significant decrease in lymphocytes, the lymphocyte to monocyte ratio (LMR), erythrocytes, hemoglobin, hematocrit (HCT), CD3+ T cells, CD3+CD4+ T cells, and CD3+CD8+ T cells were found in the severe and critically-severe group, compared with the mild and moderate group (all P < 0.05). There was no significant difference in other laboratory indicators between the mild and moderate group and the severe and critically-severe group (all P > 0.05).

### Ratio of neutrophil to T lymphocyte and neutrophil to T-lymphocyte subsets could be helpful to differentiate COVID-19 severity

**Figure 1:**
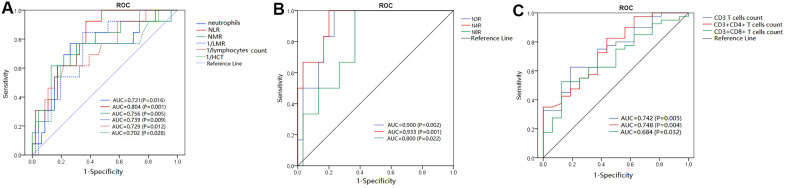
**ROC analysis of significant laboratory indicators for differentiating severe or critically- severe COVID-19 patients from mild or moderate patients.** (**A**–**C**) AUCs of key laboratory indicators, including neutrophils, NLR, NMR, LMR, HCT, N3R, N4R, N8R, CD3+ T-cell count, CD3+CD4+ T-cell count, and CD3+CD8+ T-cell count.

A receiver operating characteristic (ROC) curve analysis showed that the N4R, N3R, NLR, and N8R had potential clinical significance in differentiating severe or critically-severe patients from mild or moderate patients. Their area under ROC curve (AUC) values were 0.933, 0.900, 0.804, and 0.800, respectively (all P < 0.05). The AUCs of neutrophils, lymphocytes, CD3+ T cells, CD3+CD4+ T cells, NMR, LMR, and HCT were all less than 0.8 (all P < 0.05), and the AUC of CD3+CD8+ T cells was less than 0.7 (P < 0.05) (Figure 1). The optimal cut-off value was analyzed using the Youden Index. A diagnostic performance analysis of indicators with the best AUCs (> 0.80) was further made. It showed that under the optimal cut-off, the sensitivity (SEN) of N4R, N3R, NLR, and N8R was >90% for each parameter, and the specificity (SPE) of N4R was the best, at 80.00% (Table 3).

**Table 3:** Diagnostic performance analysis of laboratory indicators with AUC > 0.8.

**Laboratory indicators**	**AUC**	**Optimal cutoff**	**SEN**	**SPE**
N4R	0.933	14.2	100%	80.00%
N3R	0.900	7.04	100%	76.70%
NLR	0.804	3.76	92.30%	63.00%
N8R	0.800	17.76	100%	63.30%

Note: SEN: sensitivity; SPE: specificity.

### Dynamic characteristics of peripheral T-lymphocyte subsets in COVID-19 patients with different outcomes

**Figure 2:**
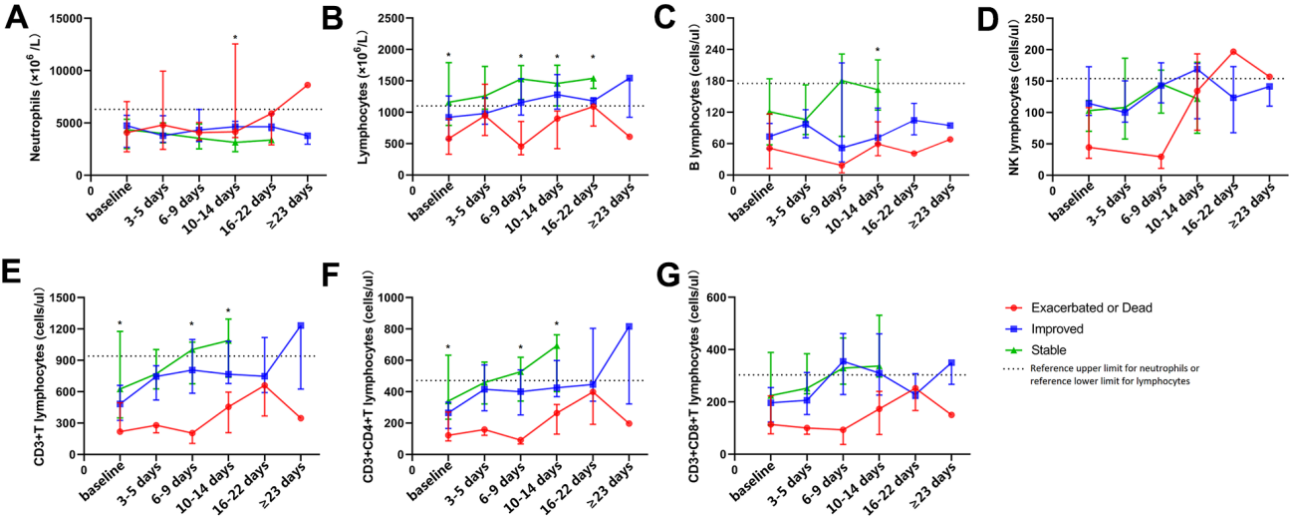
**Dynamic analysis of peripheral neutrophils, lymphocytes, and lymphocyte subsets in COVID-19 patients with different outcomes.** The dynamic changing of peripheral neutrophils (**A**), lymphocytes (**B**), B cells (**C**), NK cells (**D**), CD3+ T cells (**E**), CD3+CD4+ T cells (**F**), and CD3+CD8+ T cells (**G**) in patients with COVID-19 during hospitalization. *P < 0.05.

**Figure 3:**
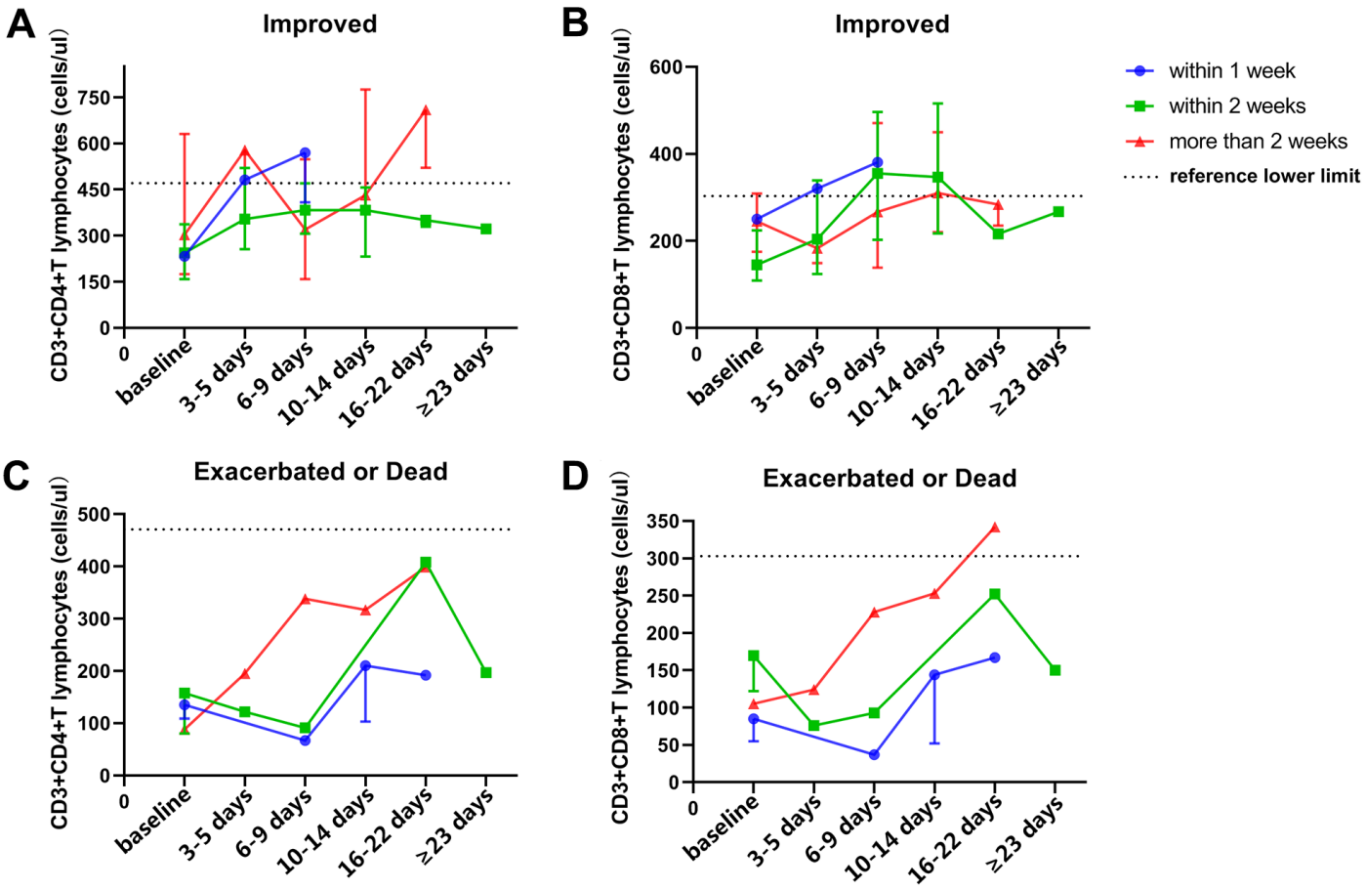
**Dynamic analysis of peripheral T-cell subsets in patients with different outcomes and endpoint times.** (**A**, **B**) dynamic characteristics of peripheral CD3+CD4+ T cells (**A**) and CD3+CD8+ T cells (**B**) in improved patients with different endpoint times after therapy. (**C**, **D**) dynamic characteristics of peripheral CD3+CD4+ T cells (**C**) and CD3+CD8+ T cells (**D**) in exacerbated or dead patients with different endpoint times after therapy.

According to disease progression, patients with COVID-19 were divided into three groups, including the stable group, improved group, and exacerbated or dead group. This study focused on elucidating the dynamic characteristics of neutrophil, lymphocyte, and T-lymphocyte subsets in COVID-19 patients with different outcomes, and it was observed that the number of lymphocytes, CD3+ T cells, and CD3+CD4+ T cells in the stable and improved groups was significantly more than that in the exacerbated or dead group at baseline, 6-9 days after baseline, and 10-14 days after baseline (P < 0.05, Figure 2). Peripheral total lymphocytes and T-cell subsets in patients with disease exacerbation were all less than the lower limit of their references during the observation period, and those in patients with a stable or improved condition gradually increased after treatment, even surpassing the upper limit of their references at 6-9 days after baseline. No dynamic characteristic of peripheral B cells or NK cells in patients with different outcomes was observed. Figure 3 shows that peripheral CD3+CD4+ or CD3+CD8+ T cells gradually increased during hospitalization, especially 6-9 days after baseline. The period of disease exacerbation could be delayed for more than 2 weeks after admission, while the period of disease improvement was shortened to 1 week after admission. On the contrary, with decreasing peripheral CD3+CD4+ or CD3+CD8+ T cells 6-9 days after baseline, COVID-19 exacerbation quickly occurred within 1 or 2 weeks of admission.

### Baseline peripheral NK cell number may correlate with SARS-CoV-2 nucleic acid expression after therapy

**Figure 4:**
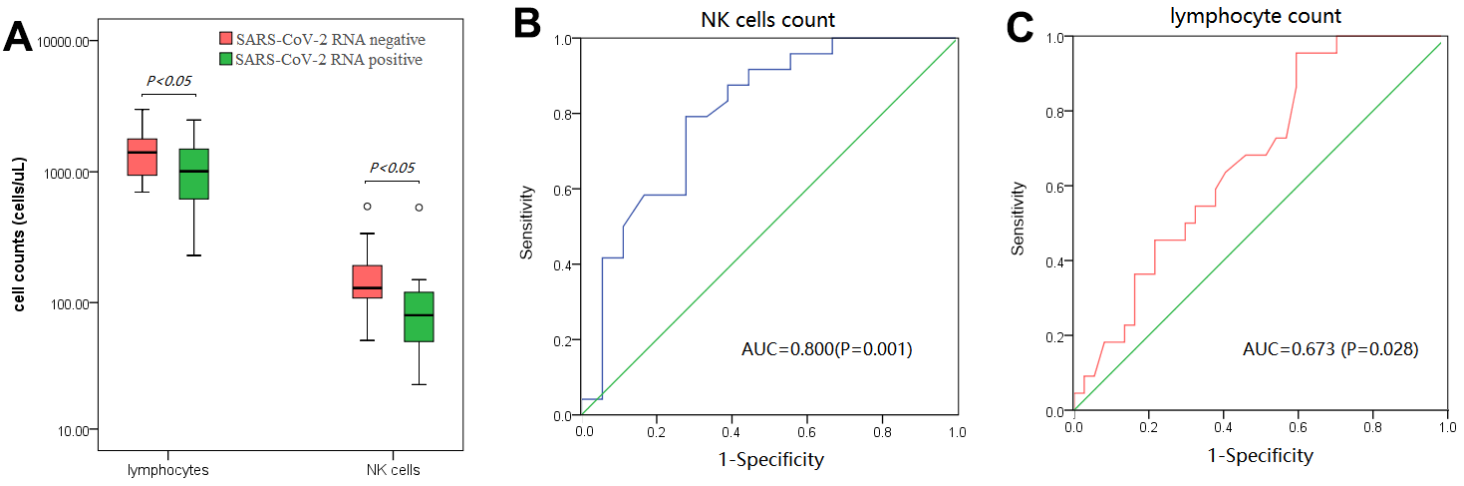
**Analysis of baseline peripheral lymphocytes and NK cells in patients with or without the SARS-CoV-2 RNA test turning negative after therapy.** (**A**) baseline peripheral lymphocytes and NK cells strikingly increased in patients whose SARS-CoV-2 RNA test turned negative after therapy. (**B**, **C**) ROC analysis of baseline peripheral NK cells (**B**) and lymphocytes (**C**) in differentiating patients whose SARS-CoV-2 RNA test turned negative after therapy.

Among 48 follow-up patients, 19 patients tested negative for SARS-CoV-2 nucleic acid after therapy. Compared with patients positive for SARS-CoV-2 after therapy, more baseline peripheral lymphocytes and NK cells could be observed in SARS-CoV-2-negative patients after therapy (P < 0.05) (Figure 4A). Additionally, there was no significant difference in other laboratory indicators between SARS-CoV-2-negative patients and SARS-CoV-2-positive patients after therapy. An ROC analysis showed that the AUCs of baseline peripheral NK cells and lymphocytes for differentiating the state of SARS-CoV-2 clearance after therapy were 0.800 and 0.673, respectively (P < 0.05) (Figure 4B, 4C). The optimal cut-off value of NK cells, defined by the Youden index, was 105 cells/μL (SEN: 79.2% and SPE; 72.2%), while the optimal cut-off value of lymphocytes was 875 cells/μL (SEN: 95.5% and SPE: 40.5%).

## DISCUSSION

COVID-19 has spread globally and was declared a pandemic in 2020. In the effort to understand this disease and the viral mechanism, studies have not only investigated its epidemiological and clinical characteristics and explored the effective therapeutics [1–3, 9] but have also begun to search for biomarkers to help clinicians in diagnosis, therapy monitoring, and the prediction of disease progression [7, 8, 10, 11]. Until now, some laboratory or imaging diagnostics of COVID-19 have been recognized, such as chest computed tomography (CT) to observe ground-glass opacity and lymphocyte profile to detect lymphopenia [1]. In the present study, 45.76% (27/59) of the COVID-19 patients exhibited lymphopenia (defined as lymphocytes < 1100 cells/μL), 15.25% (9/59) exhibited increased neutrophils, 11.86% (7/59) exhibited leukocytosis, and 8.47% (5/59) exhibited leukopenia, similar to the conclusions made by Rodriguez-Morales AJ et al. [6]. Table 1 demonstrates that in severe or critically-severe patients, the baseline number of neutrophils and lymphocyte subsets significantly increased and decreased, respectively (all P < 0.05). Laboratory indicators, especially N3R and N4R, played a significant role in auxiliary differentiating severe or critically-severe COVID-19 patients from mild or moderate patients (Figure 1), which is consistent with the conclusions of most relative studies [12]. The present study also demonstrated that when the N4R was more than 14.2, the patient could be considered to have severe or critically-severe COVID-19, with 100% SEN and 80% SPE. In COVID-19, the imbalance between neutrophils and lymphocytes, which reflects the disturbance of innate immunity and adaptive immunity induced by SARS-CoV-2, could lead to excessive inflammation without effective immune function and further cause harmful tissue damage [13]. Patients with a common viral infection, such as cytomegalovirus or Epstein-Barr virus, are characterized by increasing lymphocytes, but COVID-19 patients mainly manifest lymphopenia. To date, the pathogenesis of lymphopenia remains unclear. Some researchers have speculated that the redistribution of lymphocytes in tissues, especially in lung interstitial tissue [14], or T-cell exhaustion in immune activation due to the virus might cause lymphopenia [15, 16]. However, more evidence is required to corroborate this view. Chen N et al. demonstrated that 50% of COVID-19 patients exhibited decreased hemoglobin [3]. The present study also found that severe or critically-severe patients had mild anemia, but anemia was rarely described in other COVID-19-related studies. Anemia has not been thought of as a critical problem in COVID-19, and the reason for mild anemia in severe patients is still unclear.

In the present study, baseline peripheral B and NK cells were less abundant than the lower limit of their references in over 70% of COVID-19 patients, but no difference in B- or NK-cell count could be found between severe or critically-severe patients and mild or moderate patients. It was speculated that fewer B and NK cells in COVID-19 may influence the production of SARS-CoV-2 antibodies and clearance of the virus, but their counts were not associated with COVID-19 severity, which has been shown in other studies [7, 8]. Further analysis showed that patients with more baseline peripheral NK cells and lymphocytes were more likely to test negative for SARS-CoV-2 RNA after therapy. NK cells could play antiviral effect by indirect and direct mechanisms [17]. It was speculated that more baseline peripheral lymphocytes and NK cells may be helpful for COVID-19 patients to clear SARS-CoV-2 after therapy.

Recently, some studies have explored peripheral lymphocyte characteristics in COVID-19 patients, but they failed to conduct dynamic analyses and explore the dynamic characteristics of T-lymphocyte subsets in COVID-19 patients with different outcomes. Figure 3 shows that peripheral CD3+CD4+ and CD3+CD8+ T cells in COVID-19 patients gradually increased 6-9 days after baseline, and disease exacerbation occurred later, while disease improvement occurred earlier. On the other hand, when these cell numbers fluctuated 6-9 days after baseline, disease exacerbation could occur earlier, and improvement could occur later. Thus, the dynamic changing of T-cell subsets 6-9 days after baseline might be an important factor for the clinical evaluation of disease progression, which was consistent with the schedule of COVID-19 progression described by Huang C et al [2], and may even be an indicator for clinicians to auxiliarly assess patient outcome time.

There are some limitations in this study. First, as a retrospective study, there inevitably were missing values for clinical information or laboratory indicators, and statistical analysis was limited, that is, only dynamic characteristics and trend could be observed. Second, because it was a single-center study, the sample size was relatively small and limited. It is necessary to confirm the present results with multi-center studies. Although the dynamic characteristics of peripheral lymphocytes and T-cell subsets in COVID-19 progression were found, further research is needed to verify the conclusions.

In summary, baseline N4R and N3R can be potential biomarkers for assisting in differentiating severe or critically-severe COVID-19, and dynamically monitoring peripheral T-lymphocyte subsets, especially CD3+CD4+ and CD3+CD8+ T-cell numbers during hospitalization 6-9 days after baseline, can help clinicians to evaluate disease progression in COVID-19 patients.

## MATERIALS AND METHODS

### Subjects

Seventy-nine patients (41 males and 38 females) with an average age of 50.50 years hospitalized in the Public Health Clinical Center of Chengdu from January to February 2020 were included in this study. All patients were diagnosed with COVID-19, and disease classifications were made according to the Diagnosis and Treatment Plan of New Coronavirus Pneumonia (Trial Version 6) released by the National Health Commission and National Administration of Traditional Chinese Medicine [4]. Epidemiological history, clinical signs and symptoms, imaging results, and laboratory indicators (especially SARS-CoV-2 nucleic acid-positive) were important diagnostic standards, and for the suspected patients, continuously detecting SARS-CoV-2 nucleic acid within 2 weeks was necessary to exclude false negative results. This diagnostic protocol effectively excluded false-positive and false-negative patients. According to the guideline, patients were categorized into four subtypes: 1) mild: patients with mild symptoms without pneumonia manifestation in imaging; 2) moderate: patients with some symptoms, such as fever, respiratory tract symptoms, etc., with radiological manifestations of pneumonia; 3) severe: patients who fulfill any of the following: respiratory distress, respiratory rate ≥ 30 times/min, oxygen saturation (SpO2) ≤ 93% in resting state, partial pressure of oxygen in arterial blood/fraction of inspired oxygen (PaO2 /FiO2 ratio) ≤ 300 mmHg (1 mmHg = 0.133652 kPa), greater than 50% lesion progression in pulmonary imaging within 24 to 48 h; 4) critically severe: patients who fulfill any of the following: respiratory failure and requiring mechanical ventilation, shock, admission to the ICU with other organ failure. Cure or discharge criteria were as follows: body temperature returned to normal for more than 3 days, respiratory symptoms improved significantly, pulmonary imaging showed a significant improvement in acute exudative lesions, and two consecutive respiratory specimens tested negative for nucleic acid (sampling times were at least 24 h apart). As a retrospective study, this study was performed in accordance with the current revision of the Helsinki Declaration.

All patients were treated with basic therapeutics, including symptomatic management, oxygen inhalation, interferon atomization, antiviral therapy, or antibiotic therapy in patients with suspected bacterial infection, and mechanical ventilation and glucocorticoid treatment were used when necessary. Patients with a peripheral CD3+CD4+ T-cell count less than 350 cells/μL were treated with thymalfasin. Complete blood count and peripheral lymphocyte subsets were determined in all patients and dynamically monitored in 59 cases. The dynamic points included baseline (within 3 days of admission), 3-5 days after baseline, 6-9 days after baseline, 10-14 days after baseline, 16-22 days after baseline, and more than 23 days after baseline. All laboratory results and clinical information are described in Table 1 and Table 2, respectively.

The outcomes of all patients were recorded according to the conclusions of the clinicians from patient medical records, which were mainly dependent on patient symptoms, pulmonary CT imaging, and laboratory results (such as SpO2 and PaO2/FiO2 ratio). Of all 79 patients, 14 were mild cases, 42 were moderate cases, 12 were severe cases, and 11 were critically severe cases. After therapy, 27 patients were in a stable condition, 44 were improved, 7 were exacerbated, and 1 was dead.

### Methods

### Complete blood count and peripheral lymphocyte count analysis

Whole blood with EDTA-K2 anticoagulant was used for complete blood count analysis, which was conducted on a Mindray BC-6900 automatic blood cell analyzer (Shenzhen Mindray Bio-Medical Electronics Co., Shenzhen, China) with the corresponding reagents. Blood with lithium heparin anticoagulant was used for the peripheral lymphocyte subset count analysis, which was conducted on a Beckman Coulter Dxflex flow cytometer with Cyto-STAT tetraCHROME CD45-FITC/CD4-RD1/CD8-ECD/CD3-PC5 and Cyto-STAT tetraCHROME CD45-FITC/CD56-RD1/CD19-ECD/CD3-PC5 reagents (Beckman Coulter Company, USA). All flow cytometry data were analyzed by CytExpert for Dxflex version 2.0 software. Sample detection and quality control were performed according to the standard operating procedures and requirements of the clinical laboratory.

### Statistical analysis

All data were analyzed using the statistical software SPSS 19.0. Normality was analyzed using a Kolmogorov–Smirnov test (K-S test) and a skewness and kurtosis analysis. Data with normal distribution are described as the mean and standard deviation, and comparisons between groups were conducted using the t-test or ANOVA. Data with non-normal distribution are described as the median and interquartile, and comparisons between groups were conducted using the Mann-Whitney test. Chi-square test was used for composition ratio analysis. An ROC analysis was used to assess the clinical significance of tests in differentiating COVID-19 severity. P < 0.05 was considered statistically significant. Graphpad 8.0.1 software was used to construct dynamic graphs.
